# Microbiological Comparison of Different Sealing Materials for the Access Holes of Implant Restorations

**DOI:** 10.3290/j.ohpd.b2805461

**Published:** 2022-03-14

**Authors:** Kensuke Inoue, Hidemi Nakata, Hiromi Taninokuchi, Yuta Takahashi, Shohei Kasugai, Shinji Kuroda

**Affiliations:** a PhD Student, Department of Oral Implantology and Regenerative Dental Medicine, Division of Oral Health Sciences, Graduate School of Medical and Dental Sciences, Tokyo Medical and Dental University, Tokyo, Japan. Conceptualised the study, performed the experiments, analyzed the data, wrote the manuscript.; b Assistant Professor, Department of Oral Implantology and Regenerative Dental Medicine, Division of Oral Health Sciences, Graduate School of Medical and Dental Sciences, Tokyo Medical and Dental University, Tokyo, Japan. Conceptualised the study, supervised the study, methodology, provided funding, revised the manuscript.; c Dentist, Department of Oral Implantology and Regenerative Dental Medicine, Division of Oral Health Sciences, Graduate School of Medical and Dental Sciences, Tokyo Medical and Dental University, Tokyo, Japan. Performed the experiments, analysed the data, co-wrote the manuscript.; d Technician, Clinical Laboratory of Dental Hospital, Tokyo Medical and Dental University, Tokyo Japan. Performed the bacterial culture, analysed the data.; e Professor, Department of Oral Implantology and Regenerative Dental Medicine, Division of Oral Health Sciences, Graduate School of Medical and Dental Sciences, Tokyo Medical and Dental University, Tokyo, Japan. Conceptualised, supervised, and approved the study; methodology.; f Junior Associate Professor, Department of Oral Implantology and Regenerative Dental Medicine, Division of Oral Health Sciences, Graduate School of Medical and Dental Sciences, Tokyo Medical and Dental University, Tokyo, Japan. Supervised the study, methodology, revised the manuscript.

**Keywords:** access hole, dental prosthesis, implant restoration, microbiological evaluation, prevention of peri-implantitis, sealing material, sealing properties

## Abstract

**Purpose:**

To evaluate the performance of sealing materials used in the screw-access holes of screw-retained implant final superstructures in vivo and in vitro.

**Materials and Methods:**

Twenty-one screw-access holes in the final superstructures were randomly divided into three groups (each group, n = 7). Following disinfection and isolation, all access holes were initially filled with sterilised cotton pellets of the same weight. Depending on the group, the access holes were finally sealed with either provisional composite restorations (group A), self-curing resin for provisional sealing (group B), or acrylic resin (group C). After one month of the functional period, the inner cotton pellets were collected as bacterial reservoirs.

**Results:**

Total aerobic bacteria and total gram-negative anaerobic bacteria were measured after bacterial culture for 48 h and 72 h, respectively. In vitro evaluation of porosity using scanning electron microscopy (SEM) was also performed. Samples from superstructures sealed with provisional composite restorations showed fewer bacteria and less porosity than samples from superstructures sealed with self-curing resin for provisional sealing and acrylic resin. In this study, provisional composite restorations showed the best sealing properties. Provisional composite restorations may prevent bacterial invasion of the access holes of the final superstructures.

**Conclusion:**

In this study, provisional composite restorations showed the best sealing properties. Provisional composite restorations may prevent bacterial invasion of the access holes of the final superstructures.

Dental implant treatment is widespread in fully or partially edentulous patients,^6^ but the optimal retention system for implants is still being debated.^17,25^ There is insufficient evidence to prove differences in bone loss surrounding implant and survival rates between screw- and cement-retained implants.^2^ Studies have noted biological complications related to iatrogenic factors, such as cement excess, in cement-retained prostheses.^5,8^ Therefore, screw-retained implant prostheses might be an effective restorative method, as there is no need for cementation and the superstructure can be removed without compromising its integrity.^17^

Nevertheless, peri-implantitis can still occur in screw-retained implants and its prevention is crucial for the stability of the implant and its surrounding tissues. Peri-implantitis has a detrimental effect on the patient’s oral health, it has a multifactorial pathology, and its aetiology is not fully understood.^4,20^ However, previous studies concluded that bone loss surrounding the implant is less common in cement-retained than screw-retained implants.^7^

One possible pathological path could be related to microleakage from the sealing of screw-access holes and bacterial reservoir formation within the screw-access holes. Previous studies have shown pathogenic bacteria such as *Treponema* *denticola* inside the implant-abutment interface,^1^ and microleakage has been observed from implant-restoration access holes.^15^ Other studies have identified *Mycoplasma salivarium, Staphylococcus pasteurii, Prevotella nigrescens,* and *Prevotella melaninogenica* microleakage from screw-access holes into the inner components of implants in vitro.^3^

To our knowledge, no study has microbiologically compared the sealing properties of different outer sealing materials used in intraoral superstructures and their relationship to bacterial reservoir formation inside screw-access holes. Selecting the appropriate sealing material for screw-access holes is important, considering that anaerobic conditions may foster bacteria proliferation. Hence, access-hole sealing materials may play a significant role in preventing microleakage and, consequently, avoiding bacterial reservoirs that may be a risk factor for peri-implantitis development.

Both permanent and provisional materials can be used to seal access holes of screw-retained final superstructures. Provisional materials are easier to handle and remove from final superstructures. They are used during the first month immediately after superstructure delivery due to possible additional modifications that may necessitate the removal of superstructures. After the first month, the need for removal remains in case of fractures, biological complications, or general follow-up. Unquestionably, good aesthetic outcomes can be achieved using permanent sealing materials such as composite resins or inlays; however, superstructure integrity can be compromised during removal. Thus, many practitioners prefer provisional over permanent materials.

This study evaluated the clinical and in vitro performance of three widely used provisional sealing materials for screw-retained superstructure access holes (provisional composite restorations [CTR], self-curing resin for provisional sealing [SCRTS], and acrylic resin [AR]). We also evaluated the impact of the materials on bacterial proliferation within screw-access holes.

## Materials and Methods

This study was approved by the Ethics Committee of the University Dental Hospital of Tokyo Medical and Dental University (approval number: D2019-060) and was performed in accordance with the Declaration of Helsinki.

### Patient Selection

All patients were randomly selected from among those who underwent implant placement surgery and were about to receive the final superstructures at our institution. All surgeries followed the ‘two-stage dental implant placement’ method.

**Fig 1:**
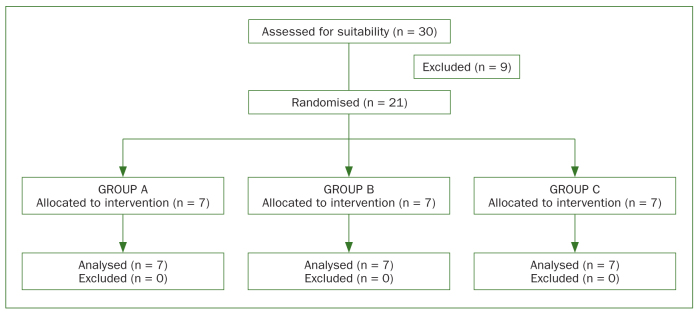
Total superstructures assessed for suitability (n = 30) and total randomised (n = 21), divided into groups A, B, and C.

Thirty screw-access holes in 11 participants (five males and six females) with an average age of 66.5 years were assessed for eligibility (Fig 1); nine screw-access holes were excluded and twenty-one screw-access holes were included. Inclusion criteria consisted of patients with a physical status classification of ‘1’ according to the American Society of Anesthesiologists, internal connection implants, Straumann SLActive implants (Straumann; Basel, Switzerland), and patients with implant superstructures placed with 35 Ncm of torque (manufacturer-recommended torque to prevent apical microleakage at its highest extent). Patients were excluded if they had periodontitis or diabetes, were undergoing radiotherapy or orthodontic treatment, were pregnant or breast-feeding, or had bruxism.

### Superstructures

The superstructures were made of Katana Zirconia (3 cases, Kuraray Noritake; Tokyo, Japan) or Sakura Zirconia (11 cases, Straumann; Basel, Switzerland). All superstructures were fabricated by the Dental Technician Laboratory in Tokyo Medical and Dental University Hospital, Tokyo, Japan.

Twenty-one screw-access holes of final superstructures with internal connection Straumann SLActive Roxolid implants (Straumann) in 9 patients were randomly analysed. After removing the provisional crown, all instruments were replaced with new, sterilised instruments. Disinfected final superstructures were filled with 0.40 g of sterilized cotton pellets (Fig 2a) and divided into three groups (each n = 7) depending on the type of sealing material used: group A, CTR (DETAX tempofill 2 inlay, DETAX; Ettlingen, Germany); group B, SCRTS (FIT SEAL , GC; Tokyo, Japan); and group C, AR (Unifast III, GC). Each material was inserted into the screw-access holes with a depth of 3 mm, as measured with a sterilised dental probe.

**Fig 2a:**
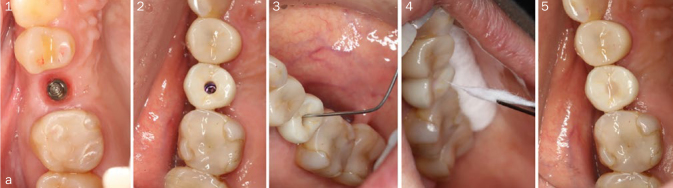
Delivery of final superstructure. (1) Before delivery of superstructure. (2) A previously disinfected final superstructure was screwed on at 35 Ncm of torque, as this is the manufacturer’s recommended torque value in order to ensure a proper sealing at the implant-abutment connection. (3) Final disinfection of the screw-access hole with hydrogen peroxide was carried out in order to ensure a disinfected environment. (4) Screw-access hole filled with 0.40 g of sterile cotton pellets previously weighed. (5) Sealed implant screw-access hole.

### Sampling

After a one-month functional period, the inner 21 cotton pellets were removed from the access holes, and immersed in 2 ml of saline solution in 15-ml conical sterile polypropylene centrifuge tubes (Nunc, Thermo Fisher Scientific; Waltham, MA, USA).

### Bacterial Culture of Intraoral Samples

Colony forming units (CFU) were counted for total aerobic (TA) bacteria in BD Trypticase Soy Agar II with 5% sheep blood (BD Diagnostics; Franklin Lakes, NJ, USA), as well as for total gram-negative anaerobic (TGNA) bacteria in Brucella HK agar containing PV (paromomycin, vancomycin) (Kyokuto Pharmaceutical Industrial; Tokyo, Japan). All sample tubes were immediately centrifuged for 60 s to detach the bacteria from the cotton pellets. The sample solution was serially diluted, and 100 μl of solution was inoculated onto the respective plates. All plates of aerobic bacteria were incubated at 37°C in an atmosphere containing 5%–10% CO_2_, and TGNA were incubated in an anaerobic incubator (Hirasawa Works; Tokyo, Japan). All procedures were performed inside a clean bench in order to prevent cross contamination.

### Bacterial Reservoirs in Cotton Pellets Extracted from Screw-access Holes

After one month of use, intraoral samples taken from screw-access holes of groups A, B, and C were cleaned three times with phosphate-buffered saline, fixed in glutaraldehyde, dehydrated with ethanol, and mounted on aluminum stubs for scanning electron microscopic (SEM) observation of bacterial reservoirs.

### Porosity of Sealing Materials In Vitro

Screw-access holes of implant-supported final restorations were obtained in vitro with either CTR, SCRTS, or AR in models with single zirconia superstructures (Fig 2b). Superstructures were analysed immediately after obturation and after seven days of immersion in artificial saliva (Saliveht Aerosol, Teijin Pharma; Tokyo, Japan).

**Fig 2b:**
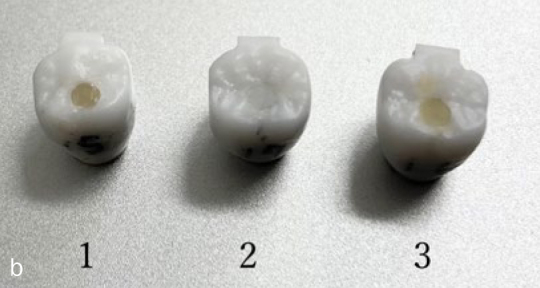
Zirconia superstructures used in SEM analysis sealed with (1) AR, (2) CTR, and (3) SCRTS.

The sealing material/superstructure interface, similar to material porosity, was observed at 60X and 100X magnifications. Optical porosity was measured using ImageJ software (version 2.0.0, National Institutes of Health; Bethesda, MD, USA). To evaluate the percentage of the material surface with pores, all measurements were reproduced in four different sections of 300 x 300 µm of 60X images after setting the scale with a known distance of 100 µm, adjusting the threshold, and analysing the particles.

### Statistical Analysis

Statistical significance (p < 0.05) was determined using one-way ANOVA with multiple comparisons for bacterial culture and surface porosity (GraphPad Prism version 8 Software; San Diego, CA, USA).

## Results

### Colony Forming Units from Bacterial Reservoirs inside the Screw-access Holes

**Figs 3:**
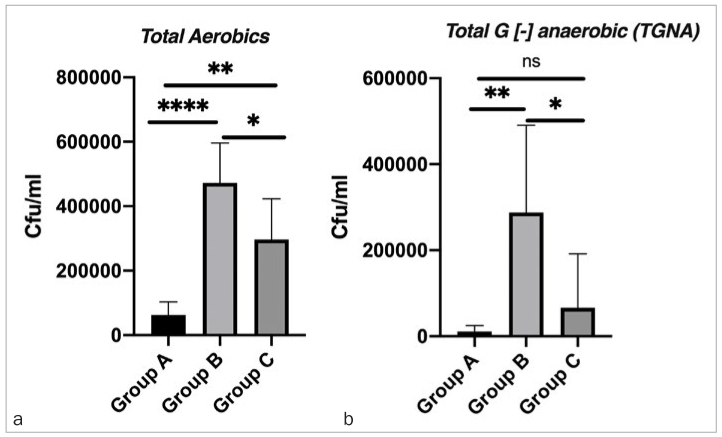
a (TA) and b (TGNA) inside screw-access holes sealed with three different sealing materials; group A: CTR; group B: SCRTS; group C: AR. Group A has statistically significantly fewer bacteria compared with the other groups, regarding both TA and TGNA. ****p < 0.0001; ***p = 0.0001 to 0.001; **p = 0.001 to 0.01; *p = 0.01 to 0.05; ns: p ≥ 0.05.

Culture techniques showed a statistically significantly lower number of both TA and TGNA bacteria in the cotton pellets from group A than in those from groups B and C (p<0.05; Figs 3A and 3B).

### SEM Analysis of Bacterial Reservoirs in Cotton Pellets

**Fig 4:**
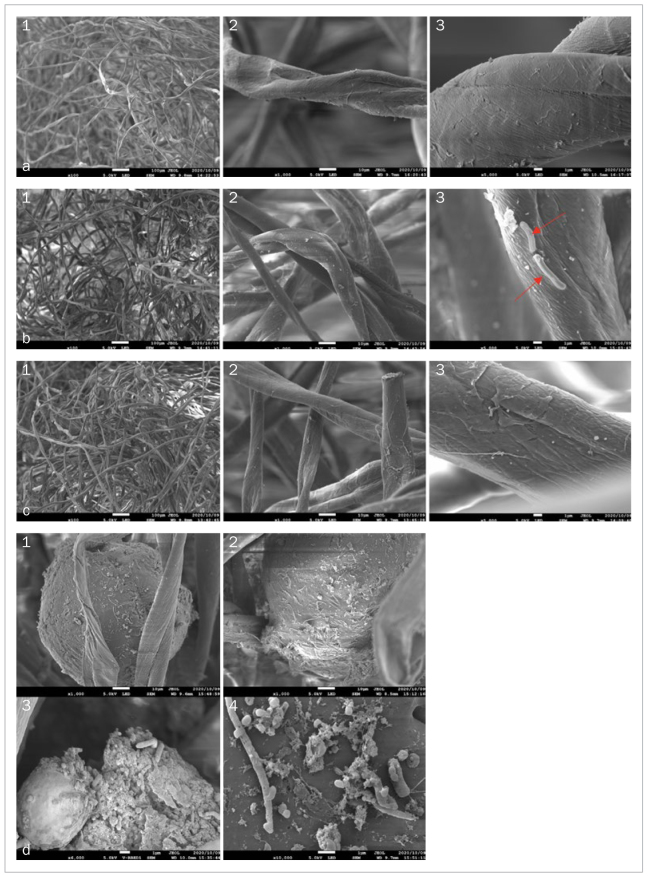
SEM image of cotton pellets inside implant screw-access holes after one month of use. Magnifications 100X, 1000X, and 5000X. a. Sealed with CTR (1) 100X; (2) 1000X; (3) 5000X: few bacteria could be seen on the surface of inserted cotton pellets. b. Sealed with SCRTS (1) 100X; (2) 1000X; (3) 5000X: numerous bacteria are visible on the surface of cotton pellets inside screw access-holes. c. Sealed with AR (1) 100X; (2) 1000X; (3) 5000X: bacteria observed on the surface of inserted cotton pellets. d. Conglomerations of bacteria forming reservoirs inside screw access-holes covered with SCRTS at (1) 100X, (2) 1000X, (3) 6000X, and (4) 10,000X.

SEM images showed more bacteria in groups B and C than in group A (Fig 4A). Group B exhibited conglomerations of bacterial reservoirs (Fig 4B), as well as a wide range of bacteria, such as cocci and bacilli, at 5000X magnification (Figs 4D to 4F).

### Porosity

**Fig 5:**
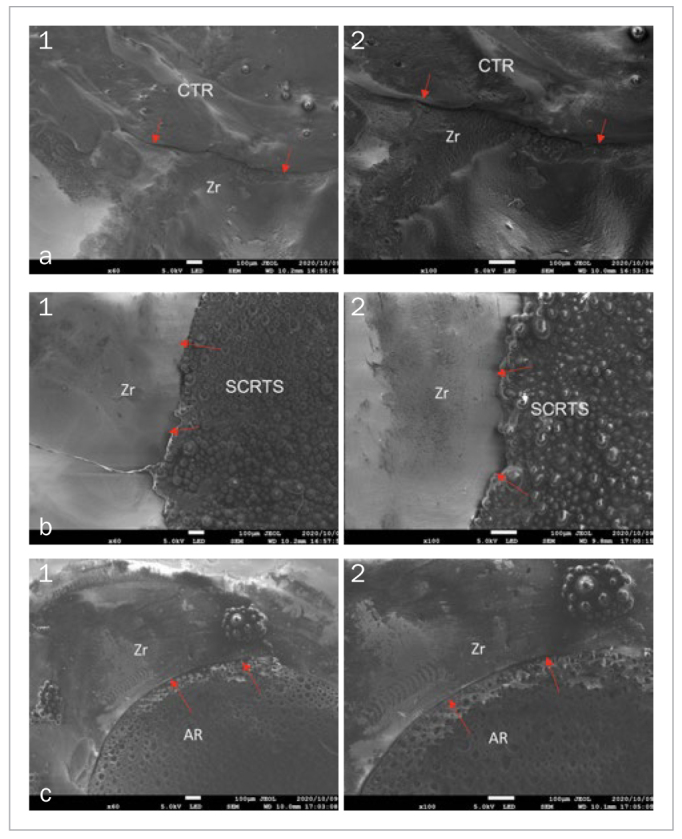
Surface porosity of materials covering screw access-holes of zirconia superstructures immediately after obturation. a. Sealed with CTR (1) 60X, (2) 100X. Interface between CTR and zirconia superstructure (red arrow). b. Sealed with SCRTS (1) 60X, (2) 100X. Interface between SCRTS and zirconia superstructure (red arrow). c. Sealed with AR (1) 60X, (2) 100X. Interface between AR and zirconia superstructure (red arrow).

**Fig 6:**
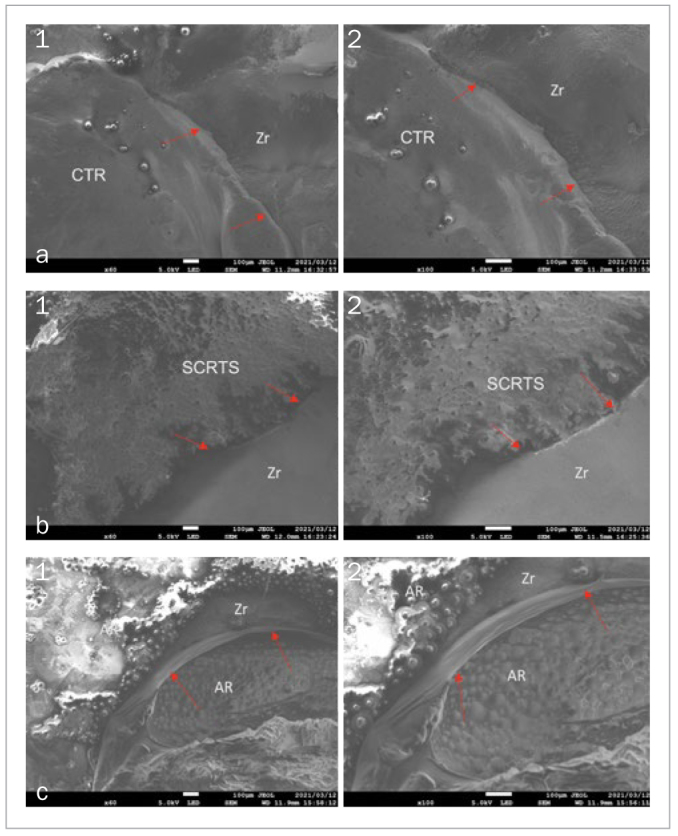
Zirconia superstructure and sealing material after 7 days of immersion in artificial saliva. A. Sealed with CTR (1) 60X, (2) 100X. Interface between CTR and zirconia superstructure (red arrow). B. Sealed with SCRTS (1) 60X, (2) 100X. Interface between SCRTS and zirconia superstructure (red arrow). C. Sealed with AR (1) 60X, (2) 100X. Interface between AR and zirconia superstructure (red arrow).

SCRTS covering screw-access holes had the highest porosity among the three materials, followed by AR. CTR was statistically significantly less porous (p<0.05), both immediately after obturation (Figs 5A to 5C) and after seven days of immersion in artificial saliva (Figs 6A to 6C). The zirconia superstructure/sealing material interface was wider for SCRTS and AR than for CTR at 60X and 100X magnification.

**Fig 7:**
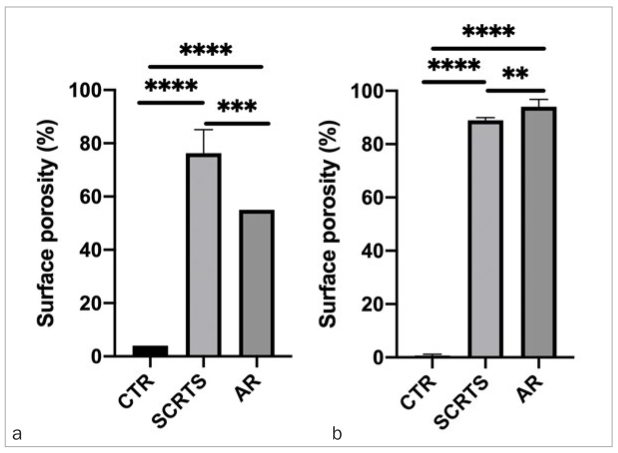
Surface porosity (percentage). (a) Immediately after obturation; (b) 7 days after immersion. ****p < 0.0001; ***p = 0.0001 to 0.001; **p = 0.001 to 0.01; *p = 0.01 to 0.05; ns: p ≥ 0.05.

## Discussion

In implant dentistry, many types of sealing or filling materials exist for screw-access holes in superstructures.^11,23^ The environmental conditions of screw-access holes (low oxygen and 37°C) are ideal for anaerobic bacteria colonisation.^13^ According to a survey in the USA, 59% of prosthodontic residency directors and 77% of restorative department chairpersons use cotton pellets to fill superstructure access holes.^18^ Some authors have found that microleakage in the implant-abutment interface is low in conical-connected implants but high in external hexagon implants.^1,12^ Thus, in this study, we only microbiologically evaluated internal connection implants, specifically in conical connection of SLActive implants. By utilising the same type of implant-abutment connection screwed with a torque value of 35 Ncm for all the implants evaluated in this study, we were able to observe how the sealing material of the final superstructures contributes to bacterial-reservoir formation inside screw-access holes.

Bacterial culture results showed the highest bacterial quantities in group B, followed by group C and then group A, indicating the superiority of CTR as a sealing material. This might be due to differences in physical properties, such as porosity and curing type. CTR is dual-curing, while AR and SCRTS are self-curing resins. Manufacturers’ instructions indicate that polymerisation occurs in 3.5 min in AR and 3 min in SCRTS. It is nearly impossible to wait for complete polymerisation due to the hydrophilic intraoral environment; this may be why self-curing resins do not show sufficient coverage properties and have higher porosity. AR and SCRTS are also exposed to masticatory forces during the early stages of the self-curing process. In contrast, light curing of CTR takes only 20 s. CTR is more durable and realistic for practitioners, and is more comfortable for patients because they do not need to keep their mouth open as long.

SEM demonstrated that CTR was less porous than AR and SCRTS. A previous study showed that composite resins modified with calcium fluoride decreased bacterial proliferation in vitro compared to those containing only fluoride compounds.^9^ In this study, we used CTR containing calcium fluoride (0.15% fluoride), which may be related to the decreased bacterial quantities in the CTR screw-access holes. This antibacterial effect may also prevent bacterial colonisation on the surface and reservoir formation inside the screw-access holes.

This study has some limitations. The influence of patient-related factors including pH and bruxism was not analysed. The intraoral pH changes constantly^21^ and varies from patient to patient,^10,16^ as do occlusal forces.^14,19^ This may influence the wear of sealing materials. We also did not evaluate flowable composite resins as sealing materials. Further research is necessary to evaluate the potential correlation between bacterial flora inside screw-access holes and the peri-implant sulcus. The performance of other materials in terms of coverage and surface porosity should also be investigated. The present authors suggest that the differences in terms of bacterial count inside the screw-access holes were not due to microleakage originating from the implant-abutment connection apically, but rather were due to microleakage originating from the access hole coronally. Nevertheless, more research is necessary to elucidate this.

## Conclusion

CTR had the best sealing properties and lowest porosity of the three materials analysed. CTR may prevent bacterial invasion, and thus the development of bacterial reservoirs, of superstructure screw-access holes.
